# Editorial: The Environment-Animal-Human Web: A “One Health” View of Toxicological Risk Analysis

**DOI:** 10.3389/fpubh.2018.00353

**Published:** 2018-12-03

**Authors:** Chiara Frazzoli, Alberto Mantovani

**Affiliations:** ^1^Department of Cardiovascular, Dysmetabolic and Aging-associated Diseases, Istituto Superiore di Sanità, Rome, Italy; ^2^Department of Food Safety, Nutrition and Veterinary Public Health, Istituto Superiore di Sanità, Rome, Italy

**Keywords:** environment health, animal health, human health, one health, toxicology, risk analysis

One Health (OH) is the conceptual and operational framework that links environment, ecosystems, and human health. Therefore, OH is a developing field, that deals with the multifaceted web of feed-backs and interactions among its components. In order to avoid “drowning into complexity,” priority issues should be identified, either for research and for risk analysis. In this book, we aimed at highlighting the importance of environment, chemical exposures and toxicological issues in the field of OH. Indeed OH has been frequently presented as an updated and more comprehensive approach to the infectious agents shared among animals and humans and the related problems, such as antibiotic resistance. Sure, topics like zoonoses feature prominently in the OH scenario which, nevertheless, includes much more environment-and-health problems. Food and environment do interact: environment influences the living organisms that produce human food and, in the meanwhile, food production outputs influence the environmental quality. As for foods of animal origin, feed materials and practices are driving components of the environment-food interactions.

The chapters address the broad spectrum of environmental and toxicological topics linked to OH, from pollution through to feeding stuffs, live animals, safe and sustainable food productions, and human risk assessment.

The dairy farm is a critical topic in the OH web: animal welfare and milk quality can reflect multiple influences, from pesticides used in crops to water quality and farm management. In the meanwhile, milk and dairy products are a major food for a large part of mankind, especially children. Dairy production is also a field for innovation in animal science. Thus, several papers deal with OH approaches in dairy farming. Three papers (“Framework to define structure and boundaries of complex health intervention systems: the ALERT project as example“ by Boriani et al.; “From invention to innovation: risk analysis to integrate One Health technology in the dairy farm” by Lombardo et al.; “Understanding seasonal changes to improve good practices in livestock management” by Martelli et al.) directly stem from the results of a national project carried in Italy in order to introduce an innovative, multi-parametric technology for the on-line monitoring of milk quality and safety. Risk analysis within OH brings about the uptake and evaluation of innovations into the agro-farming systems, as well as the need to interact with the requests of stakeholders (“Portable bio/chemosensoristic devices: innovative systems for environmental health and food safety diagnostics” by Dragone et al.).

Mycotoxins are a selected topic for OH: plant infections by fungi, modulated by agricultural practices, food production chains as well as climate changes, do produce toxins that pose risks to animal and human health. In at least one case, the Aflatoxin M1, there is an important carry-over from fungi-contaminated crops and feeds through to milk for human consumption. Mycotoxins, thus pose a significant challenge to the interdisciplinary framework of OH: approaches to risk analysis depend also from the agricultural and social scenarios (“Engaging One Health for non-communicable diseases in Africa: perspective for mycotoxins” by Ladeira et al.; “The hotspot for (global) One Health in primary food production: Aflatoxin M1 in dairy products” by Frazzoli et al.). One OH challenge is the fight against agents damaging food-producing organisms and environment through the selection of approaches that minimize the concurrent adverse side-effects. Parasytic and ectoparasytic infections, either zoonotic and non-zoonotic, are intertwined with environmental conditions and often require pharmacological treatments, especially in developing Countries. However, antiparasytic drugs can be very toxic. Efficient toxicological screens are required (“A novel strategy to predict carcinogenicity of antiparasitics based on a combination of DNA lesions and bacterial mutagenicity tests” by Liu et al.), as well as protocols that minimize the risks for farmers and the environment (“Experiences in tick control by acaricide in the traditional cattle sector in Zambia and Burkina Faso: possible environmental and public health implications” by De Meneghi et al.).

As for pesticides, they are an unavoidable support for food security. In the meanwhile, all pesticides are potentially toxic; science-based risk assessment evaluates the impact on the health of human beings (farmers and consumers), food-producing animals and on safety of life-supporting environmental goods such as drinking water. Modeling for risk assessment should consider the specific features of the agro-farming systems and allowing the identification of lower-risk options for crop protection (“Pesticides in drinking water—the Brazilian monitoring program” by Barbosa et al.; “Risk factors for non-communicable diseases in Vietnam: a focus on pesticides” by Dang et al.).

Indeed, the OH framework calls for overcoming the boundaries between environmental, biomedical and social sciences; food safety from farm to fork requires to know the “farm” (the agro-farming system) as well as the “fork” (how foods are prepared and consumed). The countries at turning point toward a more industrialized society, like several African countries, can offer scenarios of high interest for the application of OH framework (“Contaminants in Foods of Animal Origin in Cameroon: A One Health Vision for Risk Management” from Farm to Fork by Pouokam et al.). The appraisal of the scenarios is important to protect traditional agrofarming systems and the safety of their products: the development of traditional systems, such as family farming, can be, in fact, the efficient way to extract products valuable for food and nutrition security from low-quality resources. Communication and knowledge sharing with participating communities is essential (“Transdisciplinary project communication and knowledge sharing experiences in Tanzania and Zambia through a One Health lens“ by Bagnol et al.; “Safe and sustainable traditional production: the water buffalo in Asia” by Deb et al.).

Finally, this book contains thirteen papers from scientists working in institutions from eighteen Countries in Africa (Burkina Faso, Cameroon, Niger, Nigeria, South Africa, Tanzania, Zambia), Asia (Bangladesh, China, India, Philippines, Vietnam), Europe (Denmark, Italy, Portugal, Switzerland), Latin America (Brazil), and Oceania (Australia). Thus, we can confidently claim that this book, with its multiple scientific voices, provides also a contribution to global health challenges.

## Author contributions

All authors listed have made a substantial, direct and intellectual contribution to the work, and approved it for publication.

### Conflict of interest statement

The authors declare that the research was conducted in the absence of any commercial or financial relationships that could be construed as a potential conflict of interest.

